# High Expression of XRCC6 Promotes Human Osteosarcoma Cell Proliferation through the β-Catenin/Wnt Signaling Pathway and Is Associated with Poor Prognosis

**DOI:** 10.3390/ijms17071188

**Published:** 2016-07-22

**Authors:** Bin Zhu, Dongdong Cheng, Shijie Li, Shumin Zhou, Qingcheng Yang

**Affiliations:** 1 Department of Orthopedics, Shanghai Jiao Tong University Affiliated Sixth People’s Hospital, No. 600, Yishan Road, Shanghai 200233, China; zhubin.0526@163.com (B.Z.); 18817314717@163.com (D.C.); 15800553651@163.com (S.L.); 2 Institute of Orthopedics, Shanghai Jiao Tong University Affiliated Sixth People’s Hospital, No. 600, Yishan Road, Shanghai 200233, China; zhoushumin_zw@126.com

**Keywords:** XRCC6, Ku70, osteosarcoma, β-catenin/Wnt signaling pathway, proliferation

## Abstract

Increasing evidences show that XRCC6 (X-ray repair complementing defective repair in Chinese hamster cells 6) was upregulated and involved in tumor growth in several tumor types. However, the correlation of XRCC6 and human osteosarcoma (OS) is still unknown. This study was conducted with the aim to reveal the expression and biological function of XRCC6 in OS and elucidate the potential mechanism. The mRNA expression level of XRCC6 was measured in osteosarcoma cells and OS samples by quantitative transcription-PCR (qRT-PCR). The expression of XRCC6 protein was measured using Western blot and immunohistochemical staining in osteosarcoma cell lines and patient samples. Cell Counting Kit 8 (CCK8), colony-forming and cell cycle assays were used to test cell survival capacity. We found that XRCC6 was overexpressed in OS cells and OS samples compared with the adjacent non-tumorous samples. High expression of XRCC6 was correlated with clinical stage and tumor size in OS. Reduced expression of XRCC6 inhibits OS cell proliferation through G2/M phase arrest. Most importantly, further experiments demonstrated that XRCC6 might regulate OS growth through the β-catenin/Wnt signaling pathway. In conclusion, these findings indicate that XRCC6 exerts tumor-promoting effects for OS through β-catenin/Wnt signaling pathway. XRCC6 may serve as a novel therapeutic target for OS patients.

## 1. Introduction

The incidence of osteosarcoma (OS) ranks first among of all primary malignant bone tumors, predominantly affecting children and adolescence populations [1]. It often occurs in long bones, such as distal femur and proximal radius [2,3]. With the aid of effective chemotherapeutic drugs the survival rate of OS has been improved from 20% to 65% since the late 1970s [4,5]. However, treating metastatic and recurrent OS with current treatments is still limited [6]. In addition to this, the pathogenesis and etiology of osteosarcoma remain elusive [7].

X-ray repair complementing defective repair in Chinese hamster cells 6 (XRCC6) is a gene coding Ku70 protein [8]. It was reported to be involved in DNA recombination and repair, which plays a crucial role in genome stability and cell survival [9]. XRCC6 was reported as a key-element in the Non Homologous End Joining pathway and bound to DNA termini to protect DNA ends with high affinity from degradation [10]. It is hypothesized that the genetic polymorphism of XRCC6 might play an important role in tumorigenesis. The association between the expression of XRCC6 and the risk of cancer, such as lung cancer [11], glioma [12,13], hepatocellular carcinoma [14,15] and so on, has been studied by several experiments. However, the role of XRCC6 in OS is still unknown.

The β-catenin/Wnt signaling pathway plays an important role in bone development, stem cell biology and tumorigenesis [16]. Its aberrant activation has been linked to the pathogenesis of various tumors in humans. Previous studies revealed that the β-catenin/Wnt pathway contributed to OS development [17,18] and efficacy of OS chemotherapy [19]. Recently, several studies revealed that XRCC6 was a regulator of the β-catenin/Wnt signaling pathway [20,21].

In this paper, we reported, for the first time, that XRCC6 was overexpressed in human osteosarcoma. Moreover, we found that knockdown of XRCC6 expression led to growth inhibition of OS cell lines and caused β-catenin/Wnt signaling pathway dysregulation, which indicated that XRCC6 might play a crucial role in OS growth by influencing the β-catenin/Wnt signaling pathway.

## 2. Results

### 2.1. XRCC6 Is Overexpressed in Human Osteosarcoma (OS) Samples and Cell Lines

**Figure 1:**
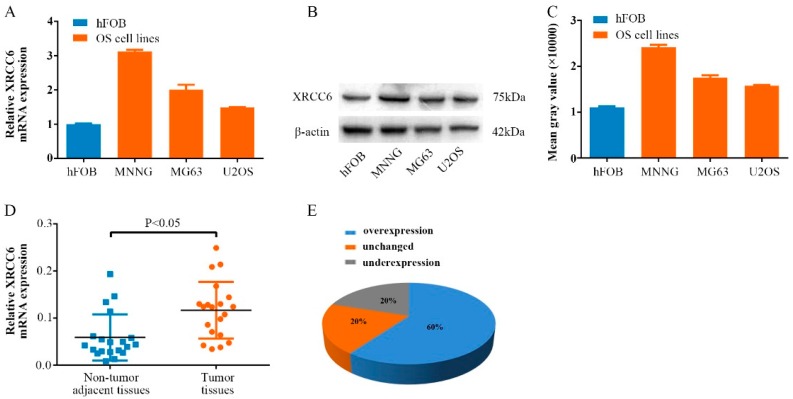
XRCC6 expression is up-regulated in osteosarcoma (OS) clinical samples and cell lines. (**A**–**C**) The mRNA and protein expression level of XRCC6 were measured by TaqMan real-time PCR and Western blot assays in OS cell lines (including MNNG/HOS, MG63 and U2OS) and human osteoblastic cell line (hFOB); (**D**,**E**) relative expression of XRCC6 was detected using qRT-PCR in 20 pairs of OS samples and their corresponding noncancerous samples. The expression of XRCC6 was overexpressed in OS tissues compared with the noncancerous tissues. Statistical analysis was performed using paired *t* test (**D**).

To determine the biological role of XRCC6 in human OS cells, we first detected the mRNA expression of XRCC6 using quantitative RT-PCR in human osteoblast cell line (hFOB) and OS cell lines (MNNG/HOS, MG63, U2OS). A higher expression of XRCC6 was observed in OS cell lines compared with human osteoblast cell lines (Figure 1A). Additionally, the same result was also found at the protein level using Western blot. Moreover, the expression of XRCC6 was detected in twenty pairs of human OS tissue samples and their adjacent non-tumorous tissue controls. We found that XRCC6 expression was significantly up-regulated in tumor tissue samples (60%) compared to the controls (Figure 1D,E).

### 2.2. Knockdown of XRCC6 Expression Inhibited OS Cell Proliferation through G2/M Phase Arrest in Vitro

**Figure 2:**
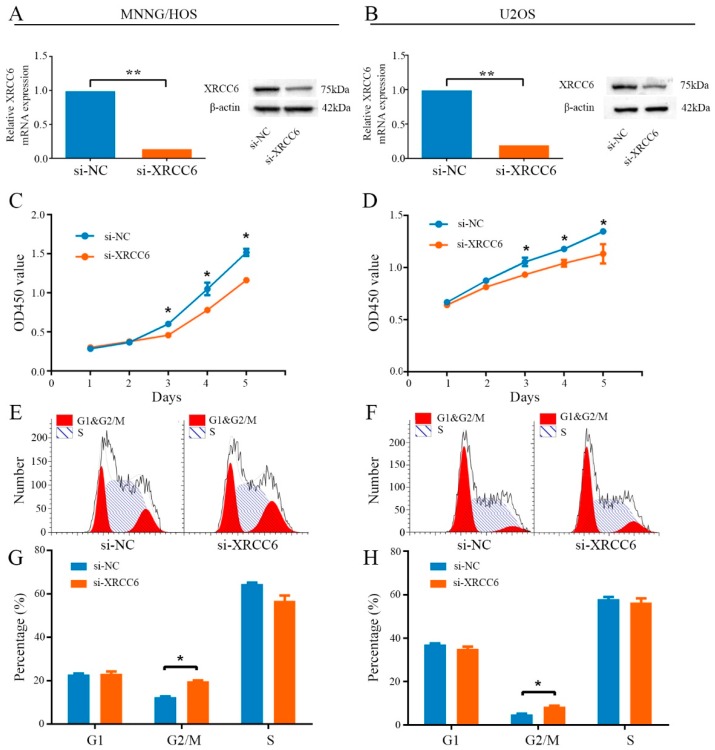
Knockdown of XRCC6 inhibited cell proliferation through G2/M phase arrest. (**A**,**B**) The expression of XRCC6 was downregulated by a targeted siRNA; (**C**,**D**) a CCK8 assay was used to detect the proliferation of MNNG/HOS cells and U2OS cells after transfection with targeted siRNA. Diagrams showing the results of a CCK-8 assay that MNNG/HOS and U2OS proliferation were inhibited by downregulating XRCC6 expression; (**E**,**G**) cell cycle profiles determined by propidium iodide (PI) staining and flow cytometry assays of MNNG/HOS transfected with si-XRCC6 or si-NC; (**F**,**H**) cell cycle profiles determined by propidium iodide (PI) staining and flow cytometry assays of U2OS transfected with si-XRCC6 or si-NC. The data are representative of three independent experiments. Error bars represent SD (Standard Deviation). * *p* < 0.05, ** *p* < 0.01 by Student’s *t* test.

In vitro experiments were carried out to evaluate the potential role of XRCC6 in tumorigenesis. MNNG/HOS and U2OS cells were transiently transfected with a targeted siRNA to decrease expression of the gene. The relative expression of XRCC6 was shown in Figure 2A,B and Figure S2. A CCK-8 assay was used to determine cell proliferation. The results showed that knockdown the expression of XRCC6 inhibited MNNG/HOS and U2OS cells proliferation (Figure 2C,D). Cell cycle changes were analyzed by flow cytometry after transfection with si-XRCC6 or si-NC. Results of cell cycle analysis revealed that inhibition of XRCC6 resulted in an elevated G2/M population in both MNNG/HOS and U2OS cells (Figure 2E–H). Thus, knockdown of XRCC6 expression may attenuate OS cell proliferation through G2/M phase arrest in vitro. However, data showed that decreased expression of XRCC6 in MNNG/HOS did not influence the ability of migration or invasion (Figure S1A,B).

### 2.3. Decreased XRCC6 Expression Impaired Colony-Forming Capacity of OS Cells

**Figure 3:**
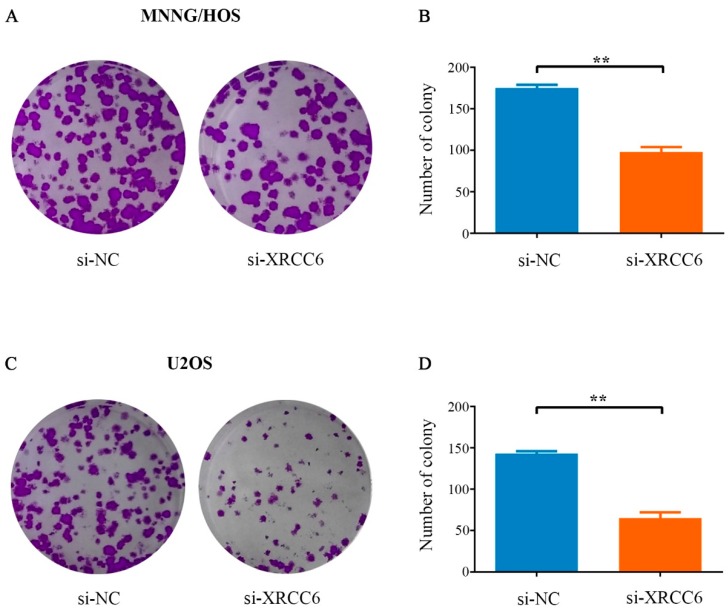
Decreased XRCC6 impaired OS cell colony-forming capacity. (**A**,**C**) Colony formation assay of MNNG/HOS cells and U2OS cells transfected with targeted siRNA or si-NC. After two weeks, cells in each well were fixed and counted. Representative photo micrographs of MNNG/HOS cells (**A**); and U2OS cells (**C**), colonies in culture plates; (**B**,**D**) significant reduction in the colony-forming efficacy in MNNG/HOS cells (**B**), and U2OS cells (**D**). Following XRCC6 knockdown. Data are expressed as mean ± SD. of three independent experiments. ** *p* < 0.01, by student’s *t* test. (Magnification: 1×).

Colony forming assay was carried out to determine the colony-forming capacity of OS cells after knockdown of XRCC6 expression. It was found that the number and the size of the colonies were both obviously decreased in the XRCC6 knockdown group in comparison with the control group (Figure 3A,C). The number of colonies was significantly reduced by 44% and 54.5% in MNNG/HOS and U2OS cells, respectively (Figure 3B,D). In conclusion, these results demonstrated that XRCC6 was important for OS cell growth.

### 2.4. The β-Catenin/Wnt Signaling Pathway Was Dysregulated by XRCC6 in OS Cells

**Figure 4:**
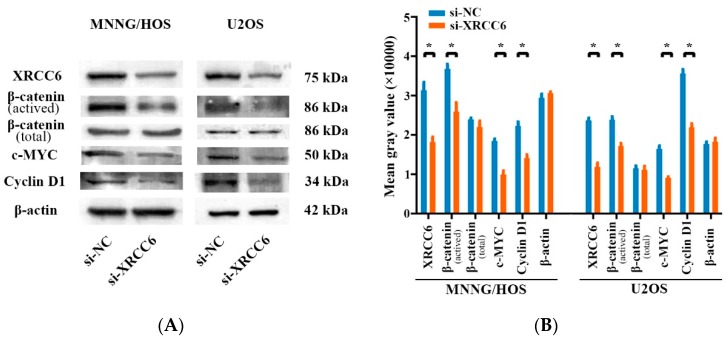
XRCC6 regulates the β-catenin/Wnt signaling pathway. (**A**) Western blot analysis of β-catenin and the downstream protein level of this pathway including c-MYC, Cyclin D1 in both MNNG/HOS and U2OS cells transfected with targeted siRNA or si-NC. Knockdown of XRCC6 expression by targeted siRNA led to reduced expression of β-catenin and the downstream protein level of this pathway in both MNNG/HOS and U2OS cells; (**B**) a densitometric analysis of the Western blotting bands was performed. * *p* < 0.05 by Student’s *t* test.

As the β-catenin/Wnt signaling pathway was widely reported in OS and correlated to tumor growth, and several studies revealed that XRCC6 was a regulator of the β-catenin/Wnt signaling pathway [20,21], we examined whether XRCC6 could influence OS cell proliferation by regulating downstream of these pathways using Western blot analysis. As shown in Figure 4, knockdown of XRCC6 expression led to a reduced expression of β-catenin and the downstream protein level of this pathway in both MNNG/HOS and U2OS cells. Taken together, XRCC6 promotes OS cell proliferation through the β-catenin/Wnt signaling pathway.

### 2.5. XRCC6 Expression Correlates to OS Clinical Stage and Tumor Size

**Figure 5:**
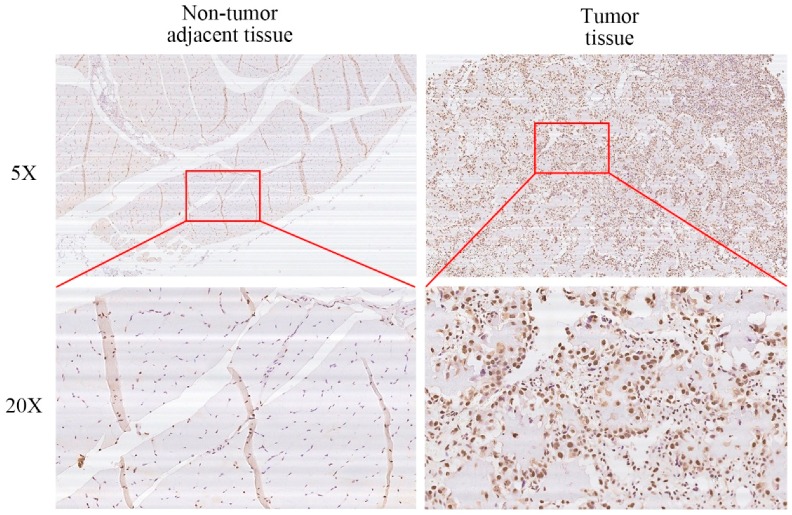
High expression of XRCC6 correlates to OS clinical stage and tumor size. Representative images of histological inspection of an OS tissue or noncancerous tissue micrographs were shown as labeled. XRCC6 was overexpressed in OS tissues compared with corresponding noncancerous tissues.

The protein levels of XRCC6 expression were detected by Immuohistochemical staining (IHC) in fifty human OS tissue samples and the corresponding non-tumorous tissue controls with the purpose to further explore the clinical relevance of the findings. The procedures were similar to those described previously [22]. Representative examples of IHC for XRCC6 in OS tissues and the noncancerous tissues are shown in Figure 5. The positive rate of IHC among OS tissues and the noncancerous tissues were 54% (27/50) and 18% (9/50), respectively. Moreover, XRCC6 expression levels were higher in human OS tissue samples than that in the corresponding non-tumorous tissues (56%, 28/50). Correlations between the XRCC6 protein expression level and clinical characteristics of OS were performed using stratified analyses and are summarized in Table 1. High expression of XRCC6 correlated positively with clinical stage (*p* < 0.05) and tumor size (*p* < 0.05). XRCC6 expression levels were negatively related to gender, age, anatomic location, tumor necrosis rate, or degree of malignancy. To conclude, these results revealed that XRCC6 was upregulated in OS tissue samples and might play an important role in OS survival and growth.

## 3. Discussion

Osteosarcoma is the most common primary malignant bone tumor with high ability of distant metastases, local recurrence and invasion [23,24]. The survival rate has increased from the 1960s to the 1980s fourfold, owing to the advances in treatments, including effective pre- and post-operative chemotherapy and wide resection of tumors [25,26]. However, the increase has stopped during the most recent three decades, and survival rates have remained unchanged [5]. Therefore, uncovering the molecular mechanisms of OS may help to identify effective therapies for OS treatment.

X-ray repair cross-complementing group (XRCC) is a family of DNA repair genes with a main function in the repair of single-strand breaks and DNA-based damage [27,28]. Multi genes in the XRCC family have been well studied in the process of carcinogenesis and have been deemed as promising genetic biomarkers. Qiao et al. revealed the potential relationship between genetic variants of XRCC1 and the susceptibility of gastric cancer in Chinese Han population, and that they might be used as molecular markers [29]. A study by Perez et al. indicated a statistical association between cervical cancer and XRCC2 [30]. Curtin et al. demonstrated that XRCC2 is crucial in colorectal cancer tumorigenesis [31].

In this article, we demonstrated that XRCC6, a member of the XRCC family, was upregulated in OS cell lines and OS tissue samples in comparison with corresponding non-tumorous tissues. In addition, we found that the expression of XRCC6 was correlated with OS Ennecking stage and tumor size according to IHC staining. The upregulation of XRCC6 has been reported in several different tumor types. Moeller et al. [32] carried out a study in a large cohort of head and neck squamous cell carcinoma (HNSCC) patients and found the overexpression of XRCC6. Marimuthu et al. [33] also reported that XRCC6 was upregulated in HNSCC cell lines through an isobaric tag for relative and absolute quantitation (iTRAQ) labeling methodology, coupled with high-resolution mass spectrometry. A study by Wang et al. revealed the increased XRCC6 expression in lung cancer [34]. However, the expression of XRCC6 in OS has not been reported before.

To further investigate whether or not the overexpression of XRCC6 has a function in OS cells, we suppressed its expression using a targeted siRNA. We found that knockdown of XRCC6 expression impairs cell proliferation ability, as well as the colony-formation ability, and led to enhancement of G2/M phase population. These results indicated that XRCC6 plays a critical role in OS cell growth and act as an oncogene in OS. The same results have been mentioned previously by other authors. Lim et al. demonstrated that XRCC6 expression was mediated by NF-κB and the high XRCC6 expression contributes to cell proliferation and carcinogenesis in gastric cancer [35]. Zhang et al. reported that XRCC6 regulates hepatocellular carcinoma cell proliferation and hepatic carcinogenesis by interacting with FOXO4 [36]. Meng et al. also showed that XRCC6 was associated with cell proliferation following cerebral hypoxia-ischemia in neonatal rats [37]. However, data showed that decreased expression of XRCC6 in MNNG/HOS did not influence migration or invasion. (Figure S1A,B)

As a evolutionarily conserved and traditional signaling pathway, the Wnt signaling pathway plays a crucial role in the regulation of multiple cellular processes, including cell fate determination, cell migration, cell polarity, and organogenesis during embryonic development and tissue homeostasis [38,39]. Several articles have reported on the association between OS and the β-catenin/Wnt signaling pathway [40,41,42]. Recent studies revealed that XRCC6 is a regulator of the β-catenin/Wnt signaling pathway in several tumor types. Puebla-Osorio et al. reported that XRCC6 was essential in tumorigenesis and proliferation in colorectal cancer and was linked to the dysregulation of the Wnt/β-catenin signaling pathway [43]. Idogawa et al. demonstrated that XRCC6 interact with TCF-4 and mediate the Wnt/β-catenin signaling pathway [44]. We found in this study that knockdown of XRCC6 expression through targeted siRNA downregulated β-catenin, c-MYC and Cyclin D1. These results suggest that XRCC6 influences OS progression by regulating the Wnt/β-catenin signaling pathway.

## 4. Materials and Methods

### 4.1. Cell Lines and Cell Culture

Two osteosarcoma cell lines were used: MNNG/HOS and U2OS. The cells were placed in a humidified atmosphere containing 5% CO_2_ with a constant temperature of 37 °C. Cells were cultured in Dulbecco’s modified Eagle’s medium (DMEM) (MNNG/HOS cells) or RPMI-1640 (U2OS cells) and supplemented with 10% fetal bovine serum (FBS) (Biowest, Kansas, MO, USA), 100 U/mL penicillin (Sigma-Aldrich, St. Louis, MO, USA), and 100 mg/mL streptomycin (Sigma-Aldrich).

### 4.2. Human Osteosarcoma Samples

Between 2013 and 2014, a total of 50 human osteosarcoma samples and the adjacent non-tumor tissues were collected during surgery at Shanghai Jiao Tong University Affiliated Sixth People’s Hospital. The tumor samples were immediately frozen in liquid nitrogen after resection and stored at −80 °C in a refrigerator. Informed consent was acquired from all patients, and the study was approved by the Ethics Committee of the Shanghai Jiao Tong University Affiliated Sixth People’s Hospital (YS-2016-064, 24 February 2016).

### 4.3. RNA Isolation and qRT-PCR Assays

Trizol Kit (Invitrogen, Carlsbad, CA, USA) and Nanodrop 2000 (Thermo Fisher Scientific, Waltham, MA, USA) were used to extract and quantify total RNA from OS cells and human OS samples. The PrimeScript RT Reagent kit (TaKaRa Biotechnology, Shiga, Japan) was used in reversely transcribed to synthesize complementary cDNA. Quantitative reversed PCR was performed using SYBR Green premix Ex Taq (TaKaRa Biotechnology) according to the manufacturer’s instructions. Expression levels were normalized using β-actin. The comparative *C*_t_ method was used to calculate the gene expression. Primer sequences used was listed below: XRCC6: Forward: TCATGGCAACTCCAGAGCAG, Reverse: AACCTTGGGCAATGTCAGGT; β-actin: Forward: TTGTTACAGGAAGTCCCTTGCC, Reverse: ATGCTATCACCTCCCCTGTGTG.

### 4.4. Cell Transfection

RNAi-max was used in the transfection of siRNA. The sequence of siRNA targeting XRCC6 was: GAAGTGACAGCTTTGAGAA. The procedure was performed according to the manufacturer’s protocol. Briefly, cells were cultured in six-well plates and the transfection was performed when the density was 40%–60% with a concentration of 50 nM of siRNA.

### 4.5. Cell Proliferation and Cell Cycle Assay

The cell viability was measured using Cell Counting Kit-8 (CCK-8) (Dojindo Molecular Technologies, Kumamoto, Japan). Briefly, cells transfected with XRCC6 siRNA were seeded in a 96-well microplate with a density of 3000 cells per well. Ten microliters of CCK8 reagent were dissolved and 100 μL RPMI-1640 (U2OS cells) or DMEM (MNNG/HOS cells) was added to each well, and a microplate reader (Mode 680) (Bio-Rad Laboratories, Hercules, CA, USA) was used to measure the absorbance at 450 nm wavelength two hours later. For cell cycle assay, cells transfected with targeted siRNA for two days were fixed in 70% ethanol and stored at −20 °C overnight. Then, the cells were stained by propidium iodide (Kaiji, Nanjing, China) at a concentration of 50 µg/mL and analyzed with a flow cytometer (BD Biosciences, San Jose, CA, USA). The assay was repeated three times. ModFit software (BD Biosciences) was used to analyze the results.

### 4.6. Migration and Invasion Assays

A 24-well plate with 8-μm pore size chamber inserts (Corning, New York, NY, USA) was utilized, according to the manufacturer’s instructions, for cell migration assays and invasion assays. For migration assays, we seeded 5 × 10^4^ OS cells in the upper chamber in 200 μL serum-free DMEM (for MNNG/HOS cells) or RPMI-1640 (for U2OS cells) per well. For invasion assays, we seeded 1 × 10^5^ OS cell in the upper chamber in 200 μL serum-free DMEM (for MNNG/HOS cells) or RPMI-1640 (for U2OS cells) per well with the Matrigel-coated membrane. Eight hundred microliters of DMEM (for MNNG/HOS cells) or RPMI-1640 (for U2OS cells), supplemented with 10% fetal bovine serum, were added in the lower chamber. Non-migrated or non-invaded cells remaining at the top surface of the inserts were removed after incubation for 13 h or 16 h at 37 °C. The cells adherent to the lower surface of the inserts were fixed and stained with 0.1% crystal violet. Cells were counted and imaged through a CKX41 inverted microscope (Olympus, Tokyo, Japan). The assay was repeated three times.

### 4.7. Colony Formation Assay

Cells transfected with targeted siRNA or siNC were seeded in a six-well plate with a concentration of 1000 cells per well and cultured in a humidified atmosphere containing 5% CO_2_ at a constant temperature of 37 °C to form colonies. Two weeks later, cells were fixed and stained with 100% methanol and 0.1% crystal violet for 20 min, separately. Colonies were air-dried and counted. The assay was repeated three times.

### 4.8. Western Blot Analysis

After extraction with a mixture of T-PER Protein Extraction Reagent (Thermo Fisher Scientific, Waltham, MA, USA), Complete Mini (Roche, Basel, Switzerland) and PhosSTOP (Roche) from cells, lysates were fractionated using sodium dodecyl sulfate polyacrylamide gel electrophoresis (SDS-PAGE) and transfected to nitrocellulose membranes (Millipore, Billerica, MA, USA). A 5% milk in phosphate buffered saline was used to block the non-specific binding sites for one hour. The membranes were then incubated at 4 °C for overnight with the following primary antibodies: XRCC6 (Proteintech, Chicago, IL, USA. 1:500), β-catenin (total) (Proteintech, 1:500), β-catenin (actived) (Bioworld Technology, St. Louis Park, MN, USA, 1:500), c-MYC (Proteintech, 1:500), Cyclin D1 (Proteintech, 1:500) or β-actin (Sigma-Aldrich, 1:20,000). The secondary antibodies were anti-rabbit IgG (Sigma-Aldrich, 1:5000) or anti-mouse IgG (Sigma-Aldrich, 1:5000). SuperSignal West Femto Maxmum Sensitivity Substrate (Thermo Fisher Scientific) was used to perform the chemiluminescence detection of the blots. Images were analyzed with ImageJ software by loading the image as a grayscale picture.

### 4.9. Immunohistochemistry (IHC)

Similar to what has been previously described [22], sections fixed with formalin and embedded with paraffin were warmed in a 60 °C oven, dewaxed in three changes of xylene, and passaged through graded ethanol (100%, 95%, and 70%) before a final wash in distilled water. The activity of endogenous peroxidase was quenched using 3% hydrogen peroxide. After blocking with BSA for 30 min, antibody against XRCC6 was used at a 1:50 dilution to incubate the section at 4 °C overnight. Counterstain was applied to all the above-mentioned slides with Gill’s Hematoxylin for one minute, then dehydrated and mounted for light microscopic evaluation. Staining intensity was classified using an established 0–3 scale, listed as follows, using systematically screening: 0, no staining; 1, staining in <1% cells; 2, staining in 1%–10% cells; 3, staining in >10% of cells. The samples classified as 0 and 1 were considered negative, while the samples classified as 2 and 3 were considered positive. In total, ten optical fields from three different sections were used for each evaluation by two experienced workmates, respectively.

### 4.10. Statistical Analysis

GraphPad Prism 5 software (Graphpad Software, Inc. La Jolla, CA, USA) was used to image all the data. The differences between the groups were compared using a two-tailed Student’s *t*-test. The correlations between the IHC of XRCC6 and the clinicopathologic parameters were determined using Perison Chi-Square and Continuity Correction Test. Data were analyzed using SPSS software (version 16.0) (IBM Corporation, New York, NY, USA). *p* < 0.05 was considered statistically significant.

## 5. Conclusions

To conclude, in this study, we provide novel evidence that XRCC6 was upregulated in OS cell lines and OS tissues. High expression of XRCC6 was correlated with clinical stage and tumor size in osteosarcoma. Reduced expression of XRCC6 inhibits OS cell proliferation. Further investigation revealed that the β-catenin/Wnt signaling pathway played an important role in the tumor-promoting effect of XRCC6. Importantly, these findings revealed that XRCC6 may become a promising new therapeutic target for the suppression of OS proliferation.

## Figures and Tables

**Table 1:** The relationship between Immunohistochemistry (IHC) of XRCC6 and their clinicopathologic parameters in 50 osteosarcoma patients.

Clinicopathologic Parameters	Number of Cases	IHC of XRCC6			
		Positive	Negative	χ^2^	*p* Value
**Age (years)**					
<20	31	17	14	0.023	0.879
≥20	19	10	9		
**Gender**					
Male	27	14	13	0.109	0.741
Female	23	13	10		
**Anatomic Location**					
Tibia/Femur	33	17	16	0.241	0.623
Elsewhere	17	10	7		
**Ennecking Stage**					
I/II	35	15	20	4.432	0.035 *
III	15	12	3		
**Tumor Size (cm^3^)**					
<50	21	7	14	6.226	0.013 *
≥50	29	20	9		
**Degree of Malignancy**					
Low	23	9	14	3.791	0.052
High	27	18	9		
**Tumor Necrosis Rate (%)**					
<90	27	16	11	0.654	0.419
≥90	23	11	12		

*p*-Value represents the probability from Perison χ-Square or Continuity Correction Test for IHC of XRCC6 expression between variable subgroups. * *p* < 0.05, which was considered to have a significant difference.

## Supplementary Materials

Supplementary materials can be found at http://www.mdpi.com/1422-0067/17/7/1188/s1.
