# Supplementary material for: High Expression of XRCC6 Promotes Human Osteosarcoma Cell Proliferation through the β-Catenin/Wnt Signaling Pathway and Is Associated with Poor Prognosis
Source: Int J Mol Sci. 2016 Jul 22;17(7):1188. doi: 10.3390/ijms17071188 (PMC4964557; doi:10.3390/ijms17071188)
Supplement: Supplementary file 1 [file ijms-17-01188-s001.pdf]

Supplementary Materials: High Expression of XRCC6 Promotes Human Osteosarcoma Cell Proliferation Through the β-Catenin/Wnt Signaling Pathway and Is Associated with Poor Prognosis

Bin Zhu, Dongdong Cheng, Shijie Li, Shumin Zhou and Qingcheng Yang

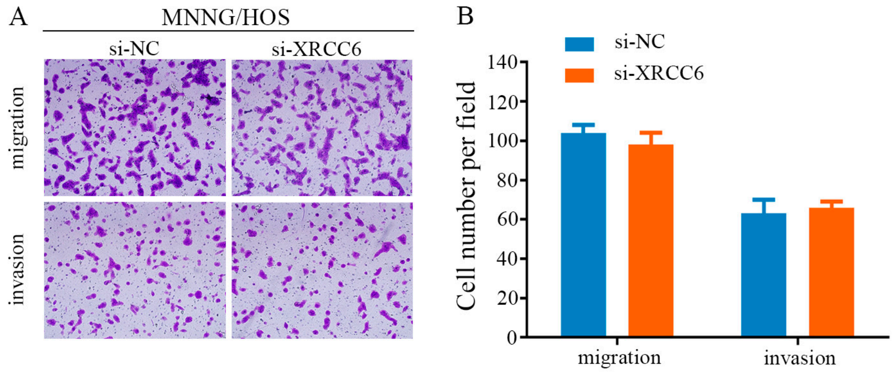

Figure S1. Knockdown of XRCC6 expression did not significantly influence migration or invasion of OS cells. (**A**,**B**) Transwell migration and invasion assays for MNNG/HOS were determined after transduction with si-NC or si-XRCC6 (Migration: 40×).

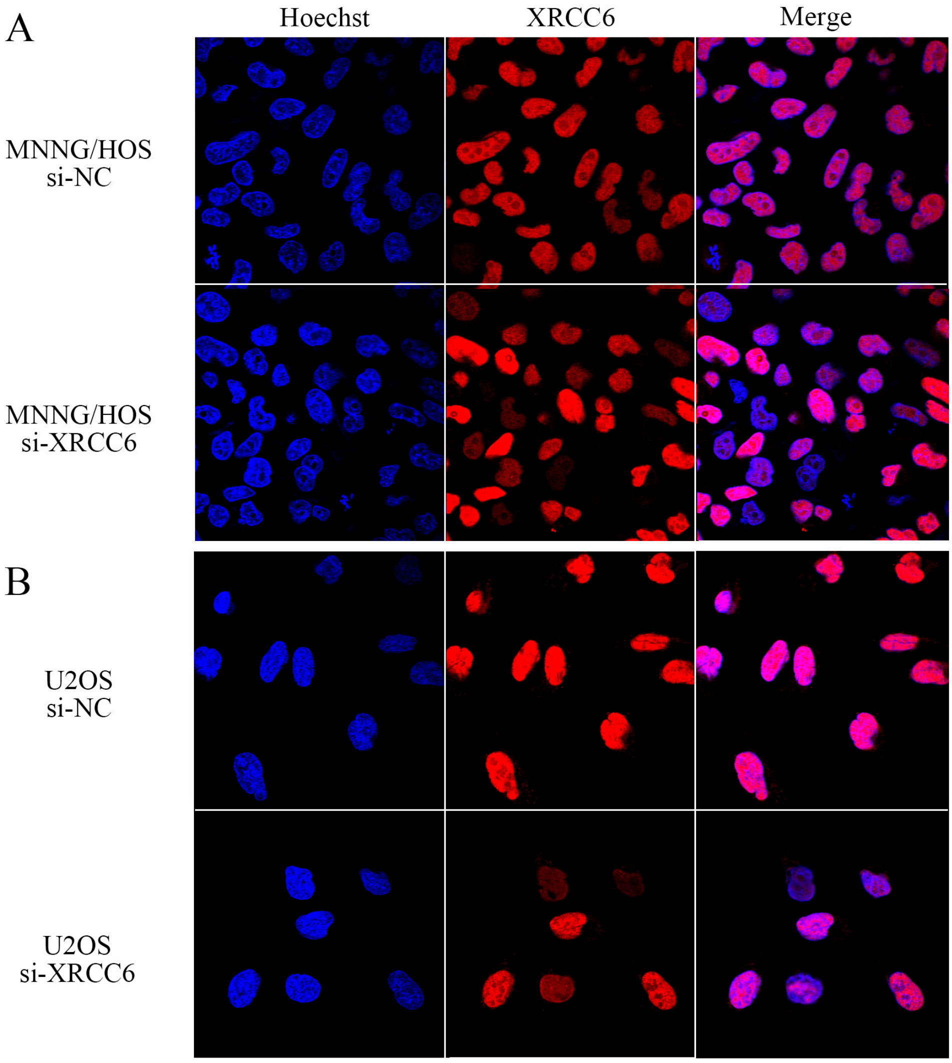

Figure S2. The expression of XRCC6 was down regulated by a targeted siRNA. (**A**,**B**) The expression of XRCC6 was detected using immunofluorescence on MNNG/HOS (**A**) and U2OS (**B**) cells after transfected with si-NC or si-XRCC6 (Migration: 200×).
